# Advances in biomarker research in multiple sclerosis

**DOI:** 10.1007/s00415-016-8062-y

**Published:** 2016-02-25

**Authors:** Sara E. Butterworth, Gillian Ingram, Neil P. Robertson

**Affiliations:** Department of Neurology, Institute of Psychological Medicine and Clinical Neuroscience, Cardiff University, Cardiff, UK

Development of novel biomarkers in multiple sclerosis (MS), needed to assess long-term prognosis, develop personalised treatment plans and facilitate earlier diagnosis, has so far been limited since potential candidates have often lacked specificity, reproducibility and accessibility. Despite considerable academic endeavour over many years, the only robust markers commonly utilised in current practice remain MRI and cerebrospinal fluid (CSF) oligoclonal bands, both of which provide limited essential prognostic information. More recently a number of alternative potential biomarkers have been proposed including chitinase-3 like 1 and neurofilament light chains both of which are based on analysis of CSF but have so far failed to be applied in clinical practice.

In this month’s journal club, we examine three recent papers investigating biomarkers in MS. The first study uses a candidate approach assessing serum B cell-activating factor (BAFF) in relation to treatment response and disease course. The second study applies a broad proteomics approach to assess extremes of disease outcome. The third study looks at genetic variants in patients on disease modifying treatments (DMT) to identify markers predictive of treatment response. These three approaches highlight the collaborative effort and extensive biobank resources required for biomarker discovery in this field.

## Changes in blood B cell-activating factor (BAFF) levels in multiple sclerosis: a sign of treatment outcome

Multiple sclerosis has traditionally been considered a T cell mediated disease; however, there is increasing awareness of the contribution of active humeral factors. BAFF is a cytokine that acts as a ligand for several tumour necrosis factor receptors and is encoded by the TNFSF13B gene. It is a potent B cell activator which plays a role in the proliferation and differentiation of B cells and is necessary for normal immunity. Excessive levels of BAFF result in high antibody production and have been linked to a number of autoimmune conditions. BAFF is also produced by astrocytes in normal brain tissue with higher levels of production in activated astrocytes. Previous studies have also shown upregulation of BAFF in MS lesions to levels usually observed in lymphoid tissue (normally ten times that of brain tissue). Increased levels of BAFF have also been demonstrated in the CSF of MS patients with secondary progressive MS and relapse and inhibition of BAFF in animal models reduces disease severity. This study aimed to examine plasma BAFF levels in relation to the clinical course of relapsing remitting MS (RRMS), relapse, steroids and disease modifying treatments (DMT).

This study included 170 patients with RRMS (50 patients with stable disease, 94 recruited during relapse and 26 during a period of remission but who went on to have a relapse within study period) and 2 controls groups with 49 healthy controls and 38 controls with acute lower back pain. Sixty-three percent of patients were receiving DMTs during the study with a mean study follow-up period of 2.3 years.

Plasma BAFF levels were significantly higher in the MS group compared to healthy controls but not to back pain controls. Within MS subgroups, patients in the stable group had significantly higher BAFF levels than those in relapse, with no significant difference in the remission group. There was no correlation of plasma BAFF with age, gender, disability prior to study entry or disease duration. In addition, levels of plasma BAFF did not change in the period following a relapse. BAFF levels were significantly higher in patients treated with Interferon-β (IFNβ) and immunosuppressant medications (mitoxantrone and cyclophosphamide) but not glatiramer acetate (GA). Plasma BAFF levels were highest in stable patients on treatment and lowest in relapsing patients on no treatment.

### Comment

So far plasma BAFF levels in MS have not demonstrated sufficient specificity or sensitivity to be of value as a clinical biomarker. Furthermore plasma BAFF is known to be raised in a number of inflammatory conditions as well as back pain, which may well be a confounder not accounted for sufficiently in this study. In this study, although elevated in stable disease there was a large degree of overlap between groups and also a number of distinct outliers with very high BAFF levels. No longitudinal data was available to assess the value of plasma BAFF in predicting of disease course, or future disability; and although elevated after DMTs, this was not predictive of clinical response. The results in this study are in conflict with some previous data but overall further reduce the likelihood of BAFF offering utility as a clinical biomarker.

Kannel K et al. (2015) PLoS One 10(11):e0143393.

## Serum proteomics in multiple sclerosis disease progression

An useful biomarker in MS would be one that allows early identification of patient subsets with defined outcomes in order to guide personalised treatment plans. However, despite multiple attempts, an accessible and reproducible biomarker of disease course has remained elusive. Proteomics is an attractive methodology to identify potential biomarkers as it provides a broad, unbiased approach. This proof-of principle study employed mass spectrometry to examine serum, as a convenient potential biomarker substrate source in aggressive and benign MS subgroups.

This study compared two extremes of the RRMS disease spectrum; benign (expanded disability status scale (EDSS) <3 after 20 years) and aggressive (EDSS >6 within 10 years of onset), with seven patients in each subgroup and no control group. Samples were retrieved from patients before use of DMTs, at various time points in their disease course. In order to identify and quantify the proteins of interest, two separate iTRAQ-MALDI-TOF/TOF experiments were undertaken. Mass spectrometry identified 108 proteins; 25 proteins were pre-filtered as a result of negligible quantities or a significant difference between males and females. The remaining 83 proteins were run on 2 iTRAQ experiments to identify proteins with 5 or more peptides. The results from the two runs were then put into the Elastic Net Model and 11 proteins were identified that significantly differed between the two MS subgroups; 7 of which were higher in the aggressive subgroup and 4 in the benign subgroup. Differences in protein concentrations between groups appeared minimal; however, when viewed as a panel, a significant difference was seen between the aggressive and benign group. Proteins identified were related to inflammation, opsonisation and complement activation cascades.

### Comment

This study was successful as a proof of principle approach and clearly needs to be repeated in larger groups of patients with appropriate control groups and longitudinal samples. There is concern that the low sample numbers used in the study may have invalidated some of the statistical models used to identify the potential biomarkers. In addition inconsistencies in the disease subgroups such as gender and timing of sample retrieval highlights some of the difficulties in conducting this type of study and it is clear that collaborative, multi-centred studies with prospective longitudinal data and standardised sample processing will be required to validate this approach. It is also of interest to note that a panel of markers was felt likely to be more informative than a single marker.

Tremlett H et al. (2015) J Proteomics 118:2–11.

## A pharmacogenetic study implicates SLC9A9 in multiple sclerosis disease activity

Treatment response in MS is highly variable and individual therapies can stabilise disease in some patients but have no apparent effect in others. Interferon-β (IFNβ) is a first-line DMT for patients with RRMS; however, studies reveal that a proportion of patients continue to experience at least one relapse during the first 2 years of treatment. As the number of MS DMTs increases, personalising therapy will become of increasing importance. This study evaluated genetic variation in the genome of MS patients in order to identify potential markers of clinical response.

This multi-centre collaborative genome-wide association study (GWAS) used RRMS patients treated with IFNβ or GA as a first-line DMT with 2 years of follow up data. Patients were classified according to treatment response into ‘responders’ (no evidence of disease activity clinically or on MR imaging at 2 years); ‘non-responders’ (decrease in annual relapse rate of less than 50 %, compared with 2 years before treatment or two or more T2/Gd enhancing lesions at first MRI or four or more T2/Gd enhancing lesions at second year MRI); or partial responders (not fulfilling either of the previous categories). The authors acknowledged a degree of variation in the clinical and MRI data available from each institute, which reduced statistical power and created some bias.

An initial discovery phase (*n* = 146) demonstrated an association between rs9828519^GG^ and non-response to IFNβ (*p* = 4.43 × 10^−8^) which was then confirmed with meta-analysis of the replication sets [Italy (*n* = 275), France (*n* = 325) and America (*n* = 557) (*p* = 7.78 × 10^−4^)]. There was no significant effect observed from rs9828519 variant on GA treatment response; however, the direction of the association signal was the same as seen with IFNβ. Only one gene, SLC9A9, was located in the linkage disequilibrium block containing rs9828519. The authors then explored the potential function of this gene demonstrating in vitro that in peripheral blood mononuclear cells, SLC9A9 expression is up-regulated following IFNβ stimulation with no difference between the rs9828519^AA^ and rs9828519^GG^ subgroups. In addition mRNA expression of SLC9A9 was reduced in MS subjects that experienced more relapses and a more activated lymphocyte profile and up-regulation of proinflammatory IFNγ in SLC9A9 knockdown polarized T helper cells in vitro.

### Comment

This study succeeds in demonstrating a gene associated with treatment response, although on an individual basis it would not have a large enough effect to tailor therapy. However, research into the functional mechanisms may be of value. The SLC9A9 gene encodes a sodium/hydrogen exchanger and is widely expressed in the CNS and immune cells, affecting the pH of the endosomal and golgi compartments of cells. Altered SLC9A9 expression could lead to altered glycosylation, an important mechanism regulating inflammation, implicating SLC9A9 variation in regulation of proinflammatory lymphocyte activation and thereby MS disease activity. The approach used in this study to identify genetic markers of MS phenotype is attractive, but as shown here, a collaborative effort with large numbers of patients and single hypothesis will be necessary to improve power.

Esposito F et al. (2015) Annu Neurol 78(1):115–127.

